# Clomiphene or enclomiphene citrate for the treatment of male hypogonadism: a systematic review and meta-analysis of randomized controlled trials

**DOI:** 10.20945/2359-4292-2025-0093

**Published:** 2025-10-08

**Authors:** Alexandre Hohl, Matheus Pedrotti Chavez, Eric Pasqualotto, Rafael Oliva Morgado Ferreira, Simone van de Sande-Lee, Marcelo Fernando Ronsoni

**Affiliations:** 1 Departamento de Clínica Médica, Universidade Federal de Santa Catarina, Florianópolis, SC, Brasil

**Keywords:** Clomiphene, enclomiphene, male hypogonadism, treatment

## Abstract

**Objective:**

This study aimed to evaluate the efficacy and safety of selective estrogen receptor modulators (SERMs), specifically clomiphene and enclomiphene, in treating men with functional hypogonadism.

**Materials and methods:**

A systematic search was conducted in PubMed, Embase, the Cochrane Library, and ClinicalTrials.gov for randomized controlled trials comparing SERMs with placebo, testosterone (T) gel, or human chorionic gonadotropin (hCG), up to July 2024. The primary endpoints were total testosterone (TT), follicle-stimulating hormone (FSH), and luteinizing hormone (LH). Weighted mean differences (MDs) and risk ratios (RRs) were calculated for continuous and binary endpoints, respectively, with 95% confidence intervals (CIs).

**Results:**

SERM therapy significantly improved TT (MD: 273.76 ng/dL; 95% CI: 191.87-355.66 ng/dL; *p <* 0.01; I^2^ = 89%), LH (MD: 4.66 IU/L; 95% CI: 3.37-5.94 IU/L; *p <* 0.01; I^2^ = 55%), and FSH (MD: 4.59 IU/L; 95% CI: 2.88-6.30 IU/L; *p <* 0.01; I^2^ = 68%) compared to placebo. No significant difference in TT was observed between the SERM and T gel groups. TT levels were significantly higher with SERM therapy and the combined treatment of SERM and hCG compared to hCG alone (158 *vs.* 153 *vs.* 134 ng/dL, respectively; *p <* 0.002 for both comparisons).

**Conclusion:**

SERM therapy is associated with significantly improved levels of TT, LH, and FSH in hypogonadal men compared to placebo, and significantly enhanced levels of LH and FSH compared to T gel. The findings suggest that SERM therapy effectively increases TT levels in men with functional hypogonadism and should be considered as an alternative to T gel therapy.

## INTRODUCTION

Male hypogonadism is a common medical condition characterized by reduced testosterone (T) levels ^(1)^. It is estimated to affect between 6% and 12% of men, with prevalence increasing with age, obesity, type 2 diabetes mellitus, and metabolic syndrome ^(1-3)^. Common symptoms associated with male hypogonadism include reduced libido, lack of energy, mood alterations, loss of muscle mass, and erectile dysfunction ^(4,5)^. These symptoms can negatively impact quality of life and overall health outcomes, highlighting the need for effective therapeutic interventions ^(6)^.

Testosterone replacement therapy (TRT) has traditionally been the cornerstone treatment for hypogonadism. However, while TRT increases serum T levels, it suppresses the hypothalamic-pituitary-gonadal axis, and compromised sperm production does not improve ^(7)^. Additionally, TRT is associated with significant adverse effects, such as increased prostate-specific antigen (PSA), elevated hematocrit, and alterations in serum lipid levels ^(8)^.

To address these limitations, various strategies have been explored to treat male hypogonadism with the goal of increasing testosterone production and restoring spermatogenesis. Clomiphene citrate and enclomiphene, selective estrogen receptor modulators (SERMs), have emerged as promising alternative therapeutic options. These drugs antagonize estrogen receptors in the hypothalamus, increasing the secretion of gonadotropin-releasing hormone and thereby stimulating endogenous T production ^(9,10)^. Unlike TRT, SERMs have the potential to preserve or even enhance fertility by maintaining or restoring spermatogenesis ^(9,11)^.

In recent years, the off-label use of SERMs for male hypogonadism has increased ^(12,13)^. However, there is limited evidence-based guidance supporting their use. Previous randomized controlled trials (RCTs) have shown mixed results regarding improvements in T levels, sperm production, and associated symptoms in men with hypogonadism ^(11,14-22)^. Therefore, we conducted a systematic review and meta-analysis of RCTs to evaluate the efficacy and safety of clomiphene and enclomiphene in the treatment of male hypogonadism.

## MATERIALS AND METHODS

This study adhered to the Preferred Reporting Items for Systematic Reviews and Meta-Analyses (PRISMA) guidelines ^(23)^. The protocol was registered with the Internat Prospective Register of Systematic Reviews (PROSPERO) under identifier no. CRD42024536930.

### Eligibility criteria

The analysis included studies that met the following eligibility criteria: 1) RCTs; 2) comparing clomiphene citrate or enclomiphene citrate (SERMs) to T gel, human chorionic gonadotropin (hCG), anastrozole, or placebo; 3) comprising only male adult patients with baseline TT levels of ≤ 300 ng/dL; and 4) reporting at least one specified outcome of interest. Conference abstracts, studies with no outcomes of interest, non-randomized trials, and studies that included patients with primary hypogonadism were excluded. There were no restrictions concerning the language or date of publication.

### Search strategy and study selection

Systematic searches were conducted in PubMed, the Cochrane Library, Embase, and Web of Science for records published from inception to April 2024. There were no language restrictions applied during searches or item selection. Search terms included Boolean combinations of the following and their deriva-tives: “Klostilbegit”, “Clostilbegit”, “Clomid”, “Clomide”, “Dyneric”, “Serophene”, “Gravosan”, “Clomiphene Hydrochloride”, “Hydrochloride, Clomiphene”, “Clomi-fene”, “Chloramiphene”, “Clomifen”, “Clomiphene Citra-te”, “Citrate, Clomiphene”, “clomiphene”, “enclomi-phene”, “males”, “men”, “boys”, “male”, “Hypogonadism, Hypergo-nadotropic”, “Hypergonadotropic Hypogonadism”, “Hypogonadism, Hypogonadotropic”, “Hypogonadism, Isolated Hypogonadotropic”, “Hypogonadotropic Hypo-gonadism”, “hypogonadism”. Detailed search strings are available in **Supplementary Table S1**. Additionally, the references of the included studies and systematic reviews were searched for additional eligible studies.

Two authors (AH and MPC) independently screened the titles and abstracts of all studies identified by the search strategy ^(13)^. Full-text article/study reports of all potentially relevant studies were retrieved for analysis through inclusion and exclusion criteria. Controversies about study eligibility were resolved by consensus with the senior author (MFR).

### Endpoints

The primary endpoints were total testosterone (TT), luteinizing hormone (LH), and follicle-stimulating hormone (FSH). Secondary endpoints included free testosterone (FT), dihydrotestosterone (DHT), estradiol, sperm concentration (million/mL), change from baseline in sperm concentration, the rate of men with sperm concentration < 15 million/mL, fasting blood glucose (FBG), glycated hemoglobin (HbA1c), insulin, body mass index (BMI), questionnaires, and adverse events.

### *Post-hoc* analysis

A *post-hoc* analysis was conducted to evaluate the existing safety data of SERMs. Accordingly, the criterion for study eligibility in this analysis did not include the testosterone threshold of 300 ng/dL for the diagnosis of hypogonadism, due to variations in thresholds used by studies in the existing literature.

### Data extraction and data items

Two investigators (AH and MPC) independently extracted data from the selected studies onto dedicated spreadsheets. The following study and participant characteristics were extracted: TT threshold used for hypogonadism diagnosis, follow-up duration, sample size, age, BMI, HbA1c, TT, LH, FSH, PSA; proportion of subjects with type 2 diabetes mellitus, and ADAM questionnaire score. Data presented as graphs in the original articles were extracted using the Engauge Digitizer program ^(24)^. Medians and ranges were converted to means and standard deviations ^(24)^.

Outcome data from the last follow-up in the RCTs were extracted for analysis. The retrieved data were double-checked by a third reviewer (EP), consolidated, and included in the meta-analysis software. Intervention groups were combined in studies presenting more than one SERM dose. The guidelines from the Cochrane Handbook for Systematic Review of Interventions were used for data handling and conversion ^(25)^.

### Risk of bias assessment

Two authors (EP and ROMF) independently assessed the risk of bias for each trial included. Discrepancies were resolved through consensus or consultation with a third author (MFR). The Cochrane Collaboration’s tool for assessing risk of bias in RCTs (RoB-2) was utilized for this evaluate the risk of bias in individual RCTs ^(26)^. “High risk” of bias was assigned to studies with a high risk in any domain or concerns in multiple domains; “some concerns” were identified for studies with concerns in any domain, and a “low risk” was noted otherwise.

The risk of publication bias could not be assessed via funnel plot analysis or Egger’s regression asymmetry test due to a limited number of studies for each endpoint ^(27)^. This limitation led to the conclusion that analyzing publication bias through these methods would be statistically underpowered and potentially misleading.

### Sensitivity analyses

Sensitivity analyses were performed to identify potential sources of heterogeneity in effect estimates. This included leave-one-out sensitivity analyses, where each study was sequentially removed from the meta-analyses and re-analyzing the pooled effect sizes. A reversal or loss of significance in effect size during recalculated pooled effects indicates potential imprecision attributable to influential studies ^(28)^.

In addition, random effects meta-regression explored the influence of baseline TT, BMI, and age on the pooled effects of clomiphene or enclomiphene citrate on TT, LH, and FSH.

### Evidence quality

The Grading of Recommendations, Assessment, Development, and Evaluations (GRADE) guidelines were employed to evaluate the quality of evidence ^(29)^. The endpoints that were quantitatively assessed were classified into four categories: high, moderate, low, or very low-quality evidence. These classifications were determined based on the risk of bias, inconsistency of results, imprecision, and the magnitude of the treatment effect.

### Summary of the measures

Effect sizes for continuous endpoints reported on the same scale were summarized as weighted mean differences (MDs), and those evaluated with different methods were reported with standardized mean differences (SMDs). Risk ratios (RRs) summarized endpoints of binary variables. Ninety-five percent confidence intervals (95% CIs) were estimated for all summary measures.

### Synthesis of the results

Random effects meta-analyses estimated pooled effect sizes for endpoints reported by two or more studies ^(30)^. Endpoints not quantitatively assessed were reported based on individual study results. Heterogeneity was assessed using the Cochrane Q-test and I^2^ statistics, with significance defined as *p*-values < 0.10 and I^2^ values > 25% ^(31)^.

### Trial sequential analysis

Trial sequential analysis (TSA) was conducted to determine if the cumulative evidence for the TT endpoint was adequately powered to detect a beneficial effect of the intervention with 90% power at the 0.05 significance level. The conventional boundary (with α error of 5%), the trial sequential monitoring boundaries, and the cumulative sequential z-score curve were plotted to compare SERM with placebo, and SERM with T gel groups. The required information sizes were estimated using the DerSimonian-Laird random-effects model ^(32)^.

### Software

Statistical analyses were performed using the R statistical software v. 4.4.0 (R Foundation for Statistical Computing). The Trial Sequential Analysis software (Copenhagen Trial Unit, Centre for Clinical Intervention Research, Copenhagen) was used for TSA.

## RESULTS

### Study selection and characteristics

**Figure 1:**
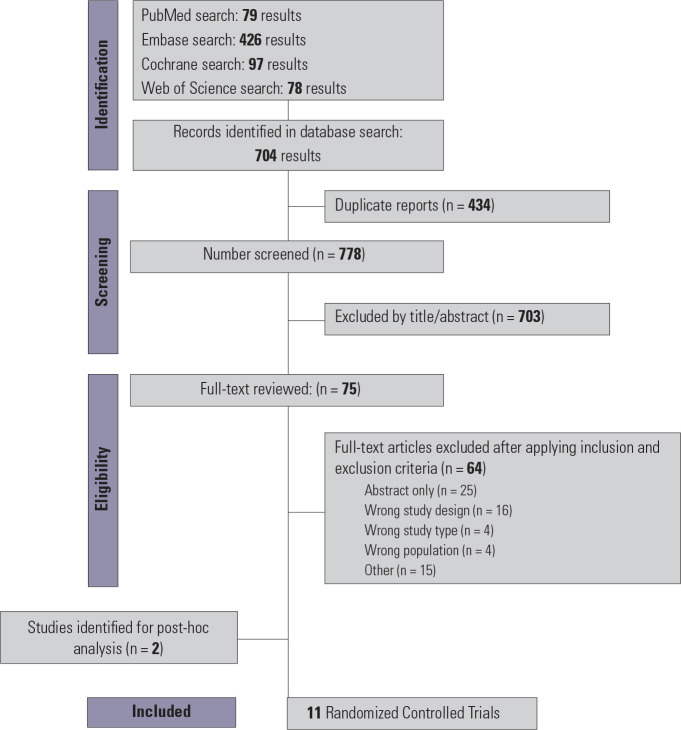
Preferred Reporting Items for Systematic Reviews and Meta-Analyses (PRISMA) flow diagram depicting the study screening and selection process.

Our search strategy identified 1,212 potential articles (**Figure 1**). After we removed duplicate records and studies that failed to meet the eligibility criteria based upon their titles and abstracts, 75 articles were thoroughly reviewed against the inclusion and exclusion criteria. Ultimately, 10 studies involving a total of 819 patients were selected for inclusion ^(11,14-22)^. Among these, two studies were incorporated into a *post-hoc* analysis ^(20,21)^. A total of 374 patients (45.7%) were assigned to SERM therapy, 133 (16.2%) to T gel, 94 (11.5%) to hCG, 13 (1.6%) to anastrozole, and 205 (25%) to a placebo. The follow-up periods varied, ranging from 2 to 30 weeks. The participants’ mean age spanned from 34 to 60.5 years. Mean baseline TT levels of 67-303 ng/dL, and mean baseline BMI of 30.5-46.4 kg/m^2^. The characteristics of the included studies are presented in **Table 1**. Further details on study features and baseline data are provided in **Supplementary Tables S2-S3**.

**Table 1:** Baseline characteristics of the included studies

Study	Study design	Patient characteristics	Intervention/control	Follow-up	Sample size, SG/CG	Age, years, SG/CG	BMI, kg/m^2^, SG/CG	TT, ng/dL, SG/CG	LH, IU/L, SG/CG	FSH, IU/L, SG/CG	T2D, SG/CG
Guay and cols. (1995)^a^	RCT, crossover, double-blind, single-center	Secondary hypogonadism; TT < 275 ng/dL; erectile dysfunction	Clomiphene citrate 50 mg (three times a week)*/* Placebo	8 w	17 (100%)/ 17 (100%)	60.5 (42-71)	NA	241±36/ 241±36	6.0±1.5/ 6.0±1.5	3.0±1.3/ 3.0±1.3	6 (35%)/ 6 (35%)
Habous and cols. (2018)	RCT, open-label, multi-center	Late-onset hypogonadism; TT < 300 ng/dL; ≥ 3 positive questions in qADAM questionnaire	Clomiphene citrate 50 mg/d*/* 5,000 IU hCG injections (twice a week)	12 w	95 (50.3%)/ 94 (49.7%)	41.8±10.4^b^	30.9/ 30.1^c^	70±23/ 64±18	NA	NA	NA
Helo and cols. (2015)^d^	RCT, double-blind, single-center	Hypogonadism; TT < 350 ng/dL; LH between 1.2 and 8.6 mIU/mL; infertility	Clomiphene citrate 25 mg/d/ Anastrozole 1 mg/d	12 w	13 (50%)/ 13 (50%)	33±3.9/ 35±6.5	32±7.5/ 33±9.8	253±17/ 248±18	3.9±0.5/ 4.8±0.5	4.2±1.7/ 9.9±1.9	NA
Kaminetsky and cols. (2013)	RCT, open-label, multi-center	Secondary hypogonadism; TT < 300 ng/dL; previous use of exogenous T	Enclomiphene citrate 25 mg/d*/* T gel	24 w (+4 w)^e^	7 (58%)/ 5 (42%)	46.0 (41-59)^b^	NA	177±77/ 138±72	2.9±1.7/ 3.4±2.1	1.6±0.7/ 3.3±0.7	NA
Kim (2016)	RCT, double-blind, multi-center	Secondary hypogonadism; TT < 300 ng/dL; overweight; low or inappropriately normal LH^f^	Enclomiphene citrate 12.5 mg/d*/* Enclomiphene citrate 25 mg/d*/* T gel 1.62%/ Placebo	16 w (+ 1 w)^e^	43 (17%)/ 42 (16%)/ 85 (33%)/ 86 (34%)	48.2±8.2^f^/ 46.2±7.8/ 47.3±8.9	33.5±4.5^g^/ 33.6±4.5/ 33.0±4.4	208±50^g^/ 219±50/ 203±45	3.7^g^/ 3.8/ 3.3	5.0^g^/ 6.1/ 5.0	NA
Pelusi and cols. (2017)^a,h^	RCT, crossover, double-blind, multi-center	Hypogonadism^i^; TT ≤300 ng/dL; BMI >30 kg/m^2^; newly diagnosed IGT or T2D	Clomiphene citrate 25 mg/d + metformin 2 g/d*/* Placebo + metformin 2 g/d	30 w	24 (100%)/ 24 (100%)	47.3±6.3/ 47.3±6.3	35.3±5.4/ 35.3±5.4	303±80/ 303±80	3.6±1.6/ 3.6±1.6	4.5±2.0/ 4.5±2.0	12 (50%)/ 12 (50%)
Pelusi and cols. (2022)^a,g^	RCT, crossover, double-blind, multi-center	Hypogonadism^b^; TT ≤ 300 ng/dL; BMI > 30 kg/m^2^; newly diagnosed IGT or T2D^c^	Clomiphene citrate 25 mg/d + metformin 2 g/d*/* Placebo + metformin 2 g/d	30 w	18 (100%)/ 18 (100%)	47.6±6.7/ 47.6±6.7	35.0±5.4/ 35.0±5.4	280±40/ 280±40	3.8±1.6/ 3.8±1.6	4.6±2.1/ 4.6±2.1	10 (56%)/ 10 (56%)
Soares and cols. (2018)	RCT, double-blind, single-center	Secondary hypogonadism; BMI > 30 kg/m^2^; symptoms in qADAM; low or inappropriately normal LH^j^	Clomiphene citrate 50 mg/d*/* placebo	12 w	39 (50%)/ 39 (50%)	35.5±7.8/ 35.6±7.8	45.5±11.3/ 47.2±9.6	226±70/ 216±72	4.3±1.8/ 5.2±2.9	4.1±2.6/ 4.7±2.5	2 (5%)/ 7 (18%)
Wiehle and cols. (2014)	RCT, double-blind, multi-center	Secondary hypogonadism; TT < 250 ng/dL	Enclomiphene citrate 12.5 mg/d*/* Enclomiphene citrate 25 mg/d*/* T gel 1%/ Placebo	12 w (+ 4 w)^e^	29 (23.4%)/ 33 (26.6%)/ 33 (26.6%)/ 29 (23.4%)	49.7±11.6/ 49.2±10.9/ 52.0±10.6/ 51.6±11.7	32.6±5.17/ 31.7±4.9/ 33.1±5.9/ 30.9±4.2	217±59/ 210±55/ 210±54/ 214±75	4.4±1.8/ 5.3±4.0/ 3.9±1.8/ 3.9±2.6	6.4±4.2/ 9.4±10.9/ 6.0±2.9/ 6.1±4.8	NA
Wiehle and cols. (2014)^d^	RCT, double-blind^k^, multi-center	Secondary hypogonadism; TT < 350 ng/dL; LH and FSH within normal ranges	Enclomiphene citrate 12.5 mg/d/ Enclomiphene citrate 25 mg/d/ Enclomiphene citrate 50 mg/d/ T gel 5 g/d/ T gel 10 g/d/ Placebo	2 w (+ 1 w)	10 (19.2%)/ 11 (21.2%)/ 11 (21.2%)/ 10 (19.2%)/ 10 (19.2%)/ 10 (19.2%)	55.2±9.1/ 53.0±14.0/ 49.2±13.1/ 51.5±14.1/ 47.1±14.1/ 50.9±14.1	NA	243±102/ 273±64/ 295±110/ 261±91/ 246±103/ 301±73	4.5±1.7/ 3.8±1.0/ 5.6±3.5/ 4.0±1.0/ 2.9±1.7/ 4.1±1.6	5.3±1.5/ 4.8±1.7/ 3.8±1.6/ 5.1±2.5/ 4.6±3.2/ 7.1±3.2	11 (21.15%)^b^

Data are means ± SD or (range) for continuous variables and n (%) for binary data. **a:** cross-over intervention; **b:** combined groups; **c:** SD not available; **d:** included in *post-hoc* analysis; **e:** follow-up interval after the end of treatment; **f:** LH < 9.4 IU/L; **g:** pooled enclomiphene citrate groups; **h:** overlapping population; i: primary causes of hypogonadism excluded; **j:** reference range 1.7-8.6 IU/L; **k:** T gel treatment had an open-label design.

BMI: body mass index; CG: control group; d: day; FSH: follicle-stimulating hormone; IGT: impaired glucose tolerance; LH: luteinizing hormone; NA: not available; qADAM: ADAM questionnaire; SD: standard deviation; SG: selective estrogen receptor modulator (SERM) group; T: testosterone; TT: total testosterone; T2D: type 2 diabetes; w: week(s).

### Primary endpoints

#### SERM vs. placebo

**Figure 2:**
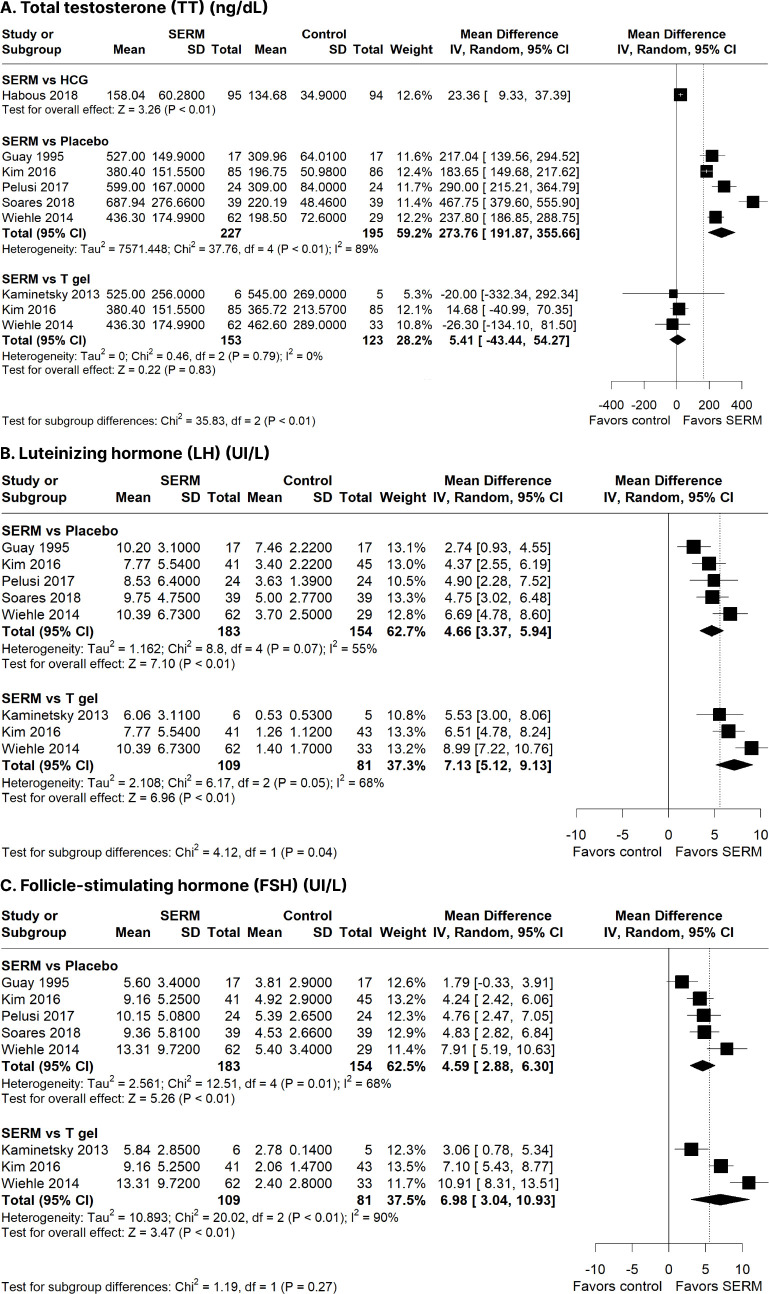
Forest plots comparing selective estrogen receptor modulators (SERMs) with placebo and testosterone (T) gel. a) Total testosterone (TT) concentration (ng/dL); b) Luteinizing hormone (LH) concentration (IU/L); c) Follicle-stimulating hormone (FSH) concentration (IU/L).

CI: confidence interval; IV: inverse variance; SD: standard deviation.

SERM therapy significantly increased TT (MD: 273.76 ng/dL; 95% CI: 191.87-355.66 ng/dL; *p* < 0.01; I^2^ = 89%; **Figure 2A**), LH (MD: 4.66 IU/L; 95% CI: 3.37-5.94 IU/L; *p* < 0.01; I^2^ = 55%; **Figure 2B**), and FSH (MD: 4.59 IU/L; 95% CI: 2.88-6.30 IU/L; *p* < 0.01; I^2^ = 68%; **Figure 2C**) compared to placebo.

#### SERM vs. T gel

There was no significant difference was observed in the TT levels of the SERM and T gel groups (MD: 5.41 ng/dL; 95% CI: -43.44-54.27 ng/dL; *p* = 0.83; I^2^ = 0%; **Figure 2A**). Nonetheless, SERM therapy was associated with significantly increased LH (MD: 7.13 IU/L; 95% CI: 5.12-9.13 IU/L; *p <* 0.01; I^2^ = 55%; **Figure 2B**) and FSH (MD: 6.98 IU/L; 95% CI: 3.04-10.93 IU/L; *p <* 0.01; I^2^ = 90%; **Figure 2C**) levels compared to T gel.

#### SERM vs. hCG

Habous and cols. ^(14)^ reported significant increases in TT with SERM therapy and combined SERM and hCG treatment compared to hCG alone (158 *vs.* 153 *vs.* 134 ng/dL, respectively; *p <* 0.002 for both comparisons). The combined treatment with SERM and hCG showed no significant difference in TT levels compared to SERM therapy alone (*p* = 0.57).

### Secondary endpoints

#### SERM vs. placebo

**Figure 3:**
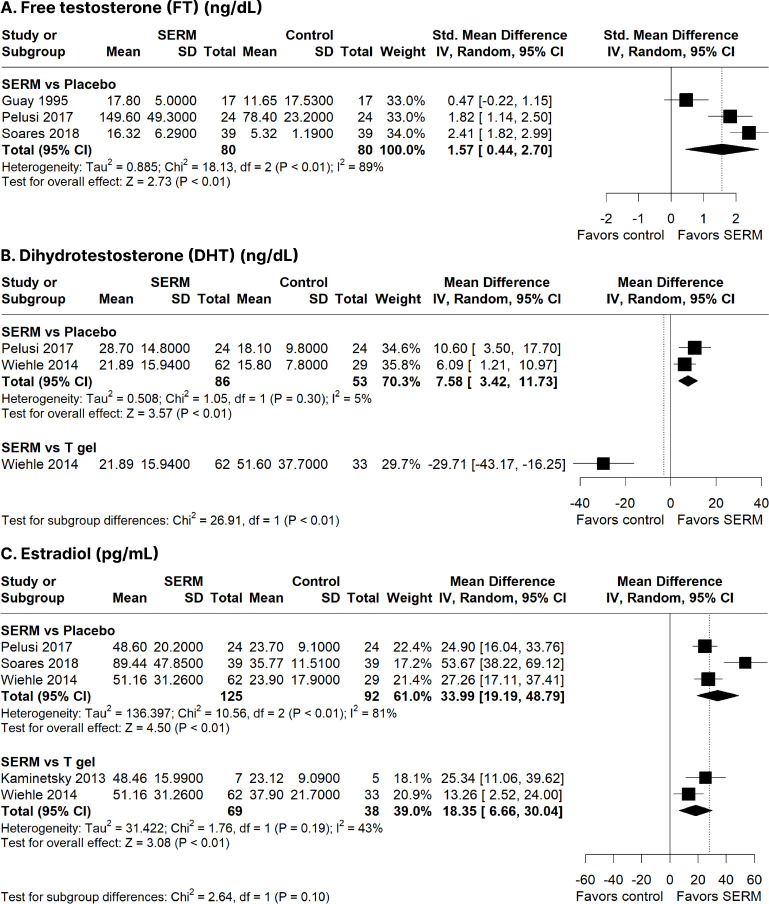
Forest plots comparing selective estrogen receptor modulators (SERMs) with placebo and testosterone (T) gel. a) Free testosterone (FT) concentration (ng/dL); b) Dihydrotestosterone (DHT) concentration (ng/dL); c) Estradiol concentration (pg/mL).

CI: confidence interval; IV: inverse variance; SD: standard deviation.

**Figure 4:**
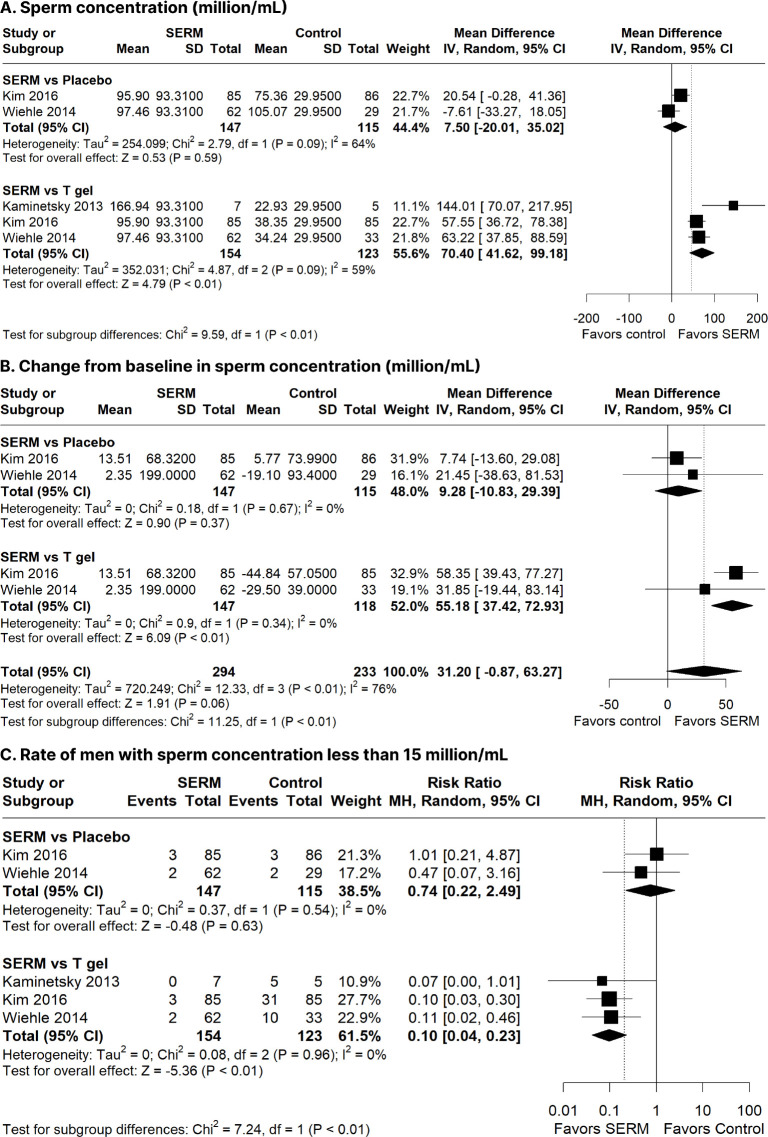
Forest plots comparing selective estrogen receptor modulators (SERMs) with placebo and testosterone (T) gel. a) Sperm concentration (million/mL); b) Change from baseline in sperm concentration (million/mL); c) Rate of men with sperm concentration less than 15 million/mL.

CI: confidence interval; IV: inverse variance; SD: standard deviation.

**Figure S1:**
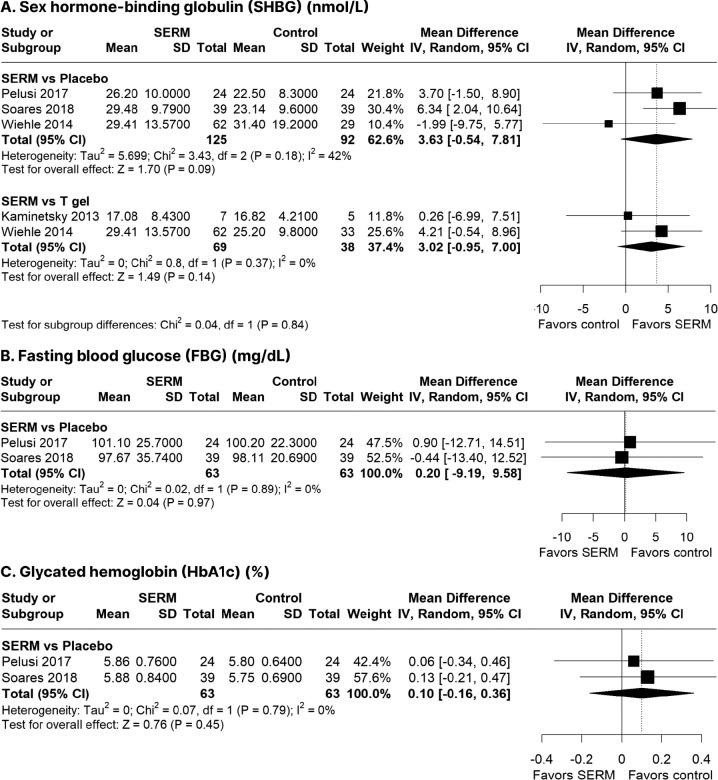
Forest plots with subgroups analysis of the comparisons between SERM therapy with placebo and testosterone gel in men with hypogonadism for **A.** Sex hormone-binding globulin; **B.** Fasting blood glucose; **C.** Glycated hemoglobin.

**Figure S2:**
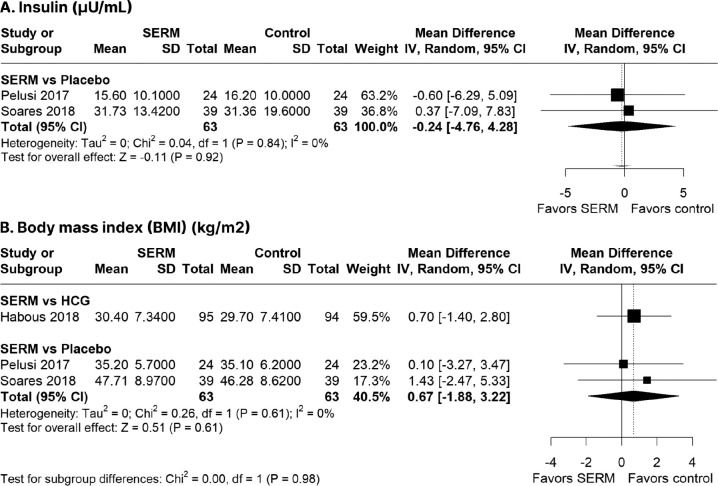
Forest plots with subgroups analysis of the comparisons between SERM therapy with placebo and testosterone gel in men with hypogonadism for **A.** insulin levels; **B.** Body mass index.

The SERM group was associated with significantly increased FT (SMD: 1.57 ng/dL; 95% CI: 0.44-2.70 ng/dL; *p <* 0.01; I^2^ = 89%; **Figure 3A**), DHT (MD: 7.58 ng/dL; 95% CI: 3.42-11.73 ng/dL; *p <* 0.01; I^2^ = 5%; **Figure 3B**), and estradiol (MD: 33.99 pg/mL; 95% CI: 19.19-48.79 pg/mL; *p <* 0.01; I^2^ = 81%; **Figure 3C**) compared to the placebo. There were no significant differences between SERM and placebo groups regarding sperm concentration (MD: 7.50 million/mL; 95% CI: -20.01-35.02 million/mL; *p* = 0.59; I^2^ = 64%; **Figure 4A**); change from baseline in sperm concentration (MD: 9.28 million/mL; 95% CI: -10.83-29.39 million/mL; *p* = 0.37; I^2^ = 0%; **Figure 4B**); rate of men with sperm concentration < 15 million/mL (RR: 0.74; 95% CI: 0.22-2.49; *p* = 0 < 0.01; I^2^ = 0%; **Figure 4C**); SHBG (MD: 3.63 nmol/L; 95% CI: -0.54-7.81 nmol/L; *p* = 0.09; I^2^ = 42%; **Figure S1A**), FBG (MD: 0.20 mg/dL; 95% CI: -9.19-9.58 mg/dL; *p* = 0.97; I^2^ = 0%; **Figure S1B**); HbA1c (MD: 0.10%; 95% CI: -0.16-0.36%; *p* = 0.45; I^2^ = 0%; **Figure S1C**); insulin (MD: -0.24 µU/mL; 95% CI: -4.76-4.28 µU/mL; *p* = 0.92; I^2^ = 0%; **Figure S2A**); and BMI (MD: 0.67 kg/m^2^; 95% CI: -1.88-3.22%; *p* = 0.61; I^2^ = 0%; **Figure S2B**).

Guay and cols. ^(11)^ reported no improvement in sexual function when comparing SERM *to* placebo, as assessed by the global sexual function index (1.6 ± 1.9 *vs.* 1.6 ± 1.7, respectively; p-value not significant) and the sexual function index questionnaires (8.8 ± 2.7 *vs.* 8.6 ± 1.9, respectively; p-value not significant). Notably, significantly improved rating scores were observed in the sexual function index among younger males compared to older men (10.0 ± 0.6 *vs.* 7.5 ± 3.6; *p <* 0.032) and in the global sexual function index for patients with diabetes or hypertension compared to those without these comorbidities (0.86 ± 1.07 *vs.* 2.8 ± 2.4, respectively; *p <* 0.018).

In the study conducted by Pelusi and cols. ^(18)^, the SERM group demonstrated a significantly higher IIEF-15 score in the sexual desire domain and a significantly lower ADAM score compared to placebo, as adjusted for pre-treatment variables. No other differences were noted.

Soares and cols. ^(19)^ found significantly decreased ADAM scores with both clomiphene and placebo. However, no group differences were observed. Similar rates of adverse events occurred in clomiphene and placebo groups, with two (5.13%) treatment discontinuations due to adverse events occurring in the placebo group, and none in the clomiphene group. Additionally, PSA values were significantly increased from baseline in the clomiphene group, though they remained within the normal range (0.62 ± 0.41 to 0.76 ± 0.48 ng/mL; *p* = 0.023). No significant differences were observed regarding International Prostate Symptom Score (*p* = 0.312) and hematocrit (*p* = 0.409).

#### SERM vs. T gel

SERM therapy yielded significantly improved levels of estradiol (MD: 18.35 pg/mL; 95% CI: 6.66-30.04 pg/mL; *p <* 0.01; I^2^ = 43%; **Figure 3C**), sperm concentration (MD: 70.40 million/mL; 95% CI: 41.62-99.18 million/mL; *p* = < 0.01; I^2^ = 59%; **Figure 4A**), change from baseline in sperm concentration (MD: 55.18 million/mL; 95% CI: 37.42-72.93 million/mL; *p* = < 0.01; I^2^ = 0%; **Figure 4B**), and rate of men with sperm concentration <15 million/mL (RR: 0.10; 95% CI: 0.04-0.23; *p* = 0 < 0.01; I^2^ = 0%; **Figure 4C**) compared with T gel. There was no significant difference between SERM and T gel regarding SHBG (MD: 3.02 nmol/L; 95% CI: -0.95-7.00 nmol/L; *p* = 0.14; I^2^ = 0%; **Figure S1A**).

Wiehle and cols. ^(22)^ found significantly lower DHT values with SERM therapy compared with T gel (enclomiphene 12.5 mg: 20.4 ± 9.1; enclomiphene 25 mg: 23.2 ± 20.2; T gel: 51.6 ± 37.7; *p <* 0.05 for both doses). Kim and cols. ^(16)^ reported a 21% rate (53 men) of adverse events possibly, probably, or definitely related to the study drugs; none of these were severe. No significant difference in the frequency of adverse events was observed among the SERM, T gel, and placebo groups. One patient (1.17%) in both the T gel and SERM groups discontinued the study due to high hematocrit/hemoglobin levels. Additionally, one man in the SERM group discontinued treatment because of elevated PSA values.

#### SERM vs. hCG

Habous and cols. ^(14)^ found significantly increased scores on the quantitative ADAM questionnaire after treatment with either clomiphene, hCG, or a combination of clomiphene and hCG (12.73 *vs.* 11.82 *vs.* 15.13, respectively). Intergroup analysis showed that scores in the combination arm were significantly improved over the other two groups (*p* < 0.01). No significant differences among groups were observed regarding BMI (30.4 *vs.* 29.7 *vs.* 31 kg/m^2^, respectively; *p* = 3.033).

#### *Post-hoc* analysis

**Table 2** summarizes the safety data and adverse events reported by each study. Helo and cols. ^(20)^ reported comparable scores on the ADAM questionnaire between the SERM and anastrozole groups (40.0 ± 1.3 *vs.* 38.0 ± 1.3; *p* = 0.634). There was one episode of pulmonary embolism with anastrozole treatment, while no serious adverse events occurred with SERM in their study. One patient (7.69%) each in the anastrozole and SERM groups discontinued treatment.

**Table 2:** Safety data and adverse events

Study	Safety data and adverse events
Helo and cols. (2015)	One treatment discontinuation occurred in the anastrozole group due to a rash, and one participant was lost to follow-up in the clomiphene citrate group. There was one episode of pulmonary embolism in a patient with a prior history of deep vein thrombosis in the anastrozole group. No serious AEs occurred in the clomiphene citrate group. Drug compliance exceeded 90%. No significant changes were observed in liver panel results or complete blood counts.
Kim and cols. (2016)	Fifty-three (21%) men experienced ad AEs related to the study drug; none were severe or serious. There was one severe AE in the placebo group, two in the T gel group, four in the 12.5 mg enclomiphene group, and two in the 25 mg enclomiphene citrate arm. All severe AEs were deemed unrelated to the treatment. There was no significant difference in treatment-related AEs between groups. The enclomiphene citrate group did not have a higher frequency of AEs compared to the placebo group. Two treatment discontinuations were observed in the 25 mg enclomiphene citrate group: one due to elevated hematocrit/hemoglobin levels and one due to high PSA levels. One treatment discontinuation occurred in the T gel group because of elevated hematocrit/hemoglobin levels. Eight treatment discontinuations were recorded in the placebo or testosterone gel arms. Two deaths occurred: one from a road accident and another in the enclomiphene citrate group due to a stroke, accompanied by multiple pre-existing risk factors.
Soares and cols. (2018)	Two members of the placebo group discontinued treatment, with one case due to a severe headache episode and another due to pneumonia-related death. There were no withdrawals related to AEs in the clomiphene citrate group. A significant increase in PSA levels within the normal range was observed in the clomiphene citrate group. No significant IPSS or hematocrit levels were observed. ALT levels showed a significant decrease in the clomiphene citrate group. Self-reported AEs were similar across both groups. Somnolence was reported by five patients in the clomiphene citrate group, while no cases were reported in the placebo group (*p* = 0.064).
Wiehle and cols. (2014)	Treatment-emergent AEs were similar across groups, occurring in 9%-20% of subjects. In the enclomiphene citrate group, AEs included one episode of mildly increased blood estradiol at 12.5 mg, one episode of mild sinus headache at 25 mg, and one episode of moderate headache at 50 mg. In the placebo groupthere was only one mild headache reported. No treatment discontinuations occurred due to AEs. No serious AEs were observed in the enclomiphene citrate group. One serious AE, severe dizziness, was noted in the topical testosterone group, but it was unrelated to the study drug. There were no significant changes in chemistry, hematology, or urinalysis attributed to either enclomiphene citrate or testosterone gel.

AE: adverse event; ALT: alanine aminotransferase; IPSS: International Prostate Symptom Score; PSA: prostate-specific antigen; T: testosterone.

In the study by Wiehle and cols. ^(21)^, adverse events were similar among the SERM, T gel, and placebo groups. In the SERM group, adverse events included one incident (3.13%) of mildly elevated blood estradiol, one mild sinus headache (3.13%), and one moderate headache (3.13%), while there was one mild headache (10%) with the placebo. No individuals discontinued treatment due to adverse events, and there were no serious adverse events related to treatment.

### Sensitivity analyses

**Figure S3:**
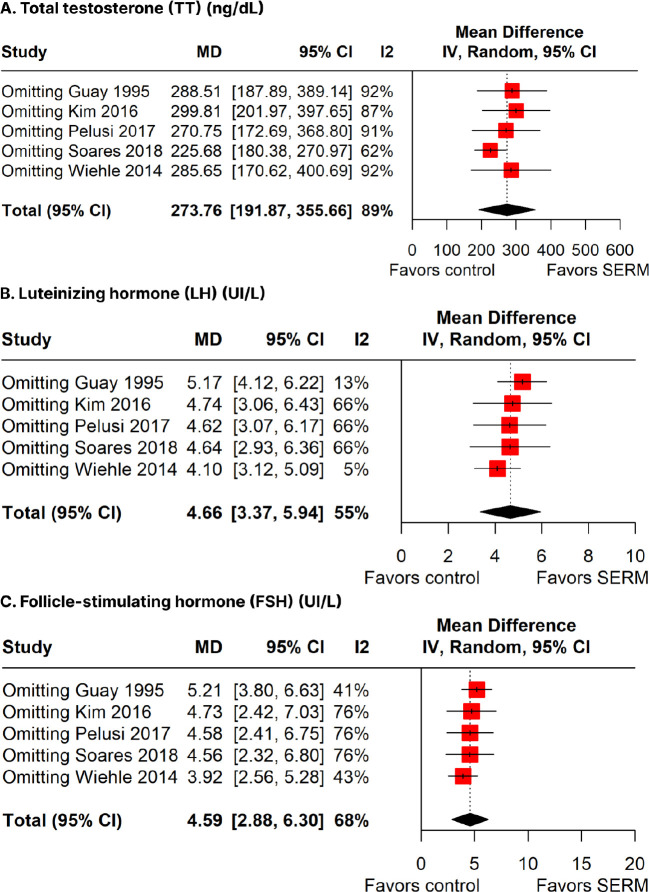
Leave-one-out sensitivity analyses for SERM therapy vs placebo regarding endpoints of **A.** Total testosterone; **B.** Luteinizing hormone; **C.** Follicle-stimulating hormone.

**Figure S4:**
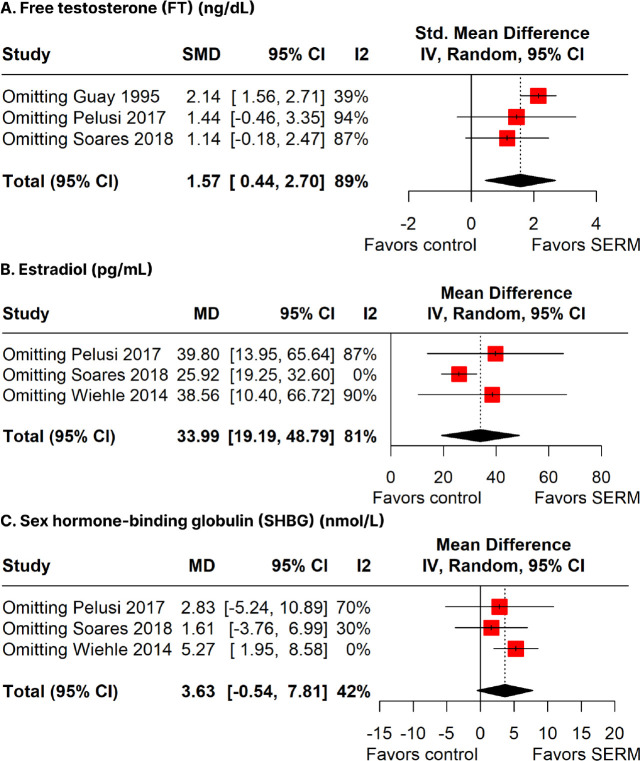
Leave-one-out sensitivity analyses for SERM therapy *vs* placebo regarding endpoints of **A.** Free testosterone; **B.** Estradiol; **C.** Sex hormone-binding globulin.

**Figure S5:**
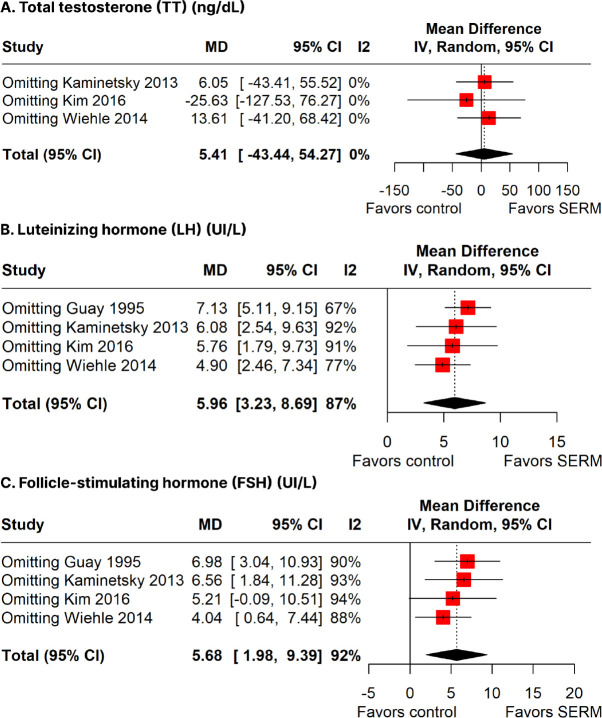
Leave-one-out sensitivity analyses for SERM therapy vs testosterone gel regarding endpoints of **A.** Total testosterone; **B.** Luteinizing hormone; **C.** Follicle-stimulating hormone.

**Figure S6:**
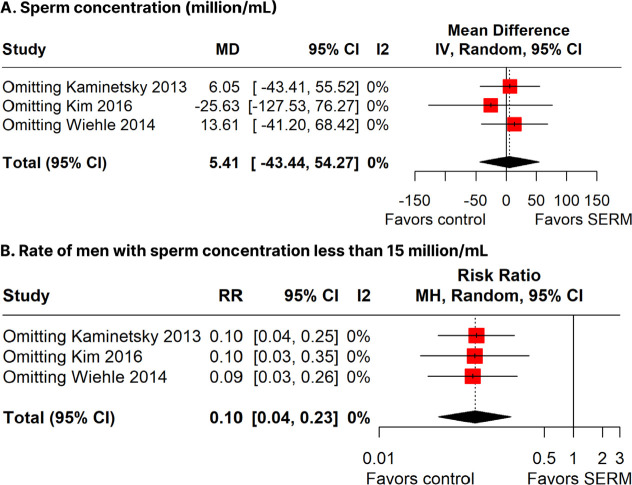
Leave-one-out sensitivity analyses for SERM therapy *vs* testosterone gel regarding endpoints of **A.** Sperm concentration; **B**. Rate of men with sperm concentration less than 15 million/mL.

No changes in p-values were observed for the overall effects of SERM *vs.* placebo in leave-one-out sensitivity analyses for the primary endpoints. However, a change from significant to non-significant for SERM therapy *vs.* T gel was observed upon the removal of the study by Kim and cols. ^(16)^ (MD: 5.21 UI/L; 95% CI: -0.09-10.51 UI/L) from the FSH endpoint. Leave one-out-sensitivity analyses are depicted in **Supplementary Figures S3-S6**.

### Meta-regression

**Figure S7:**
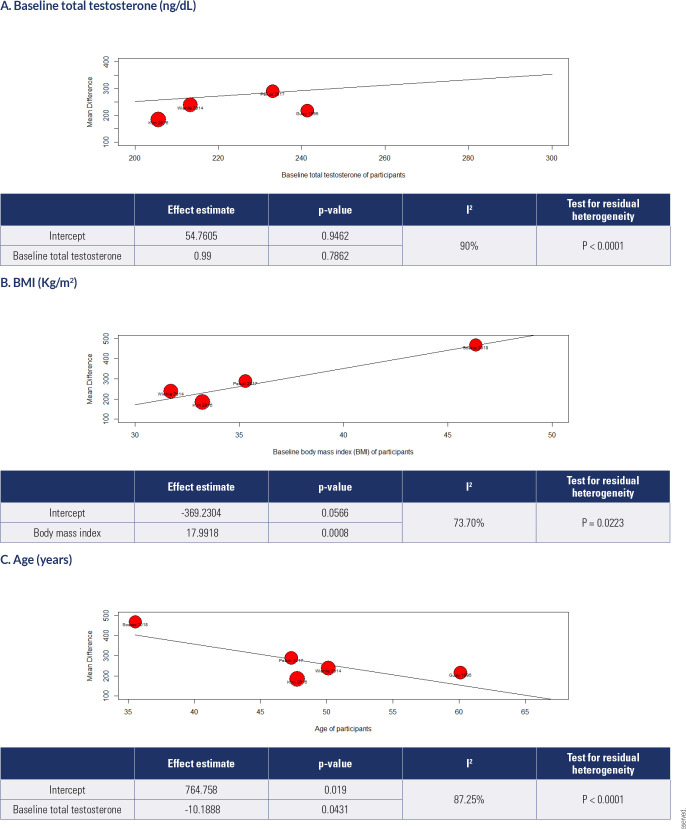
Meta-regression for SERM vs placebo assessing the impact of patients’ A. Baseline total testosterone; B. Body mass index; and C. Age on the total testosterone endpoint.

**Figure S8:**
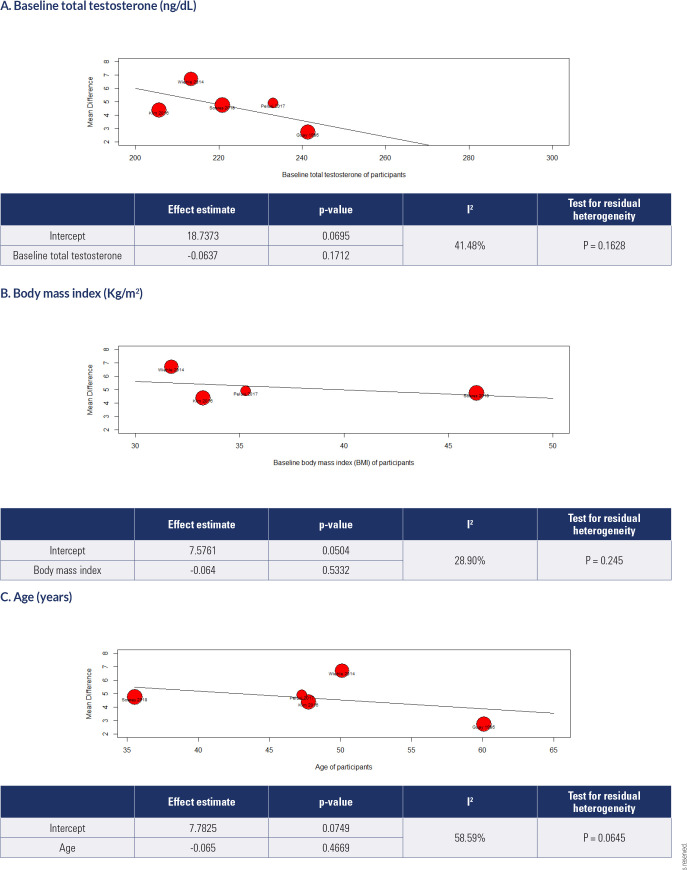
Meta-regression for SERM therapy *vs* placebo assessing the impact of patients’ **A.** Baseline total testosterone; **B.** Body mass index; and **C.** Age on the luteinizing hormone endpoint.

**Figure S9:**
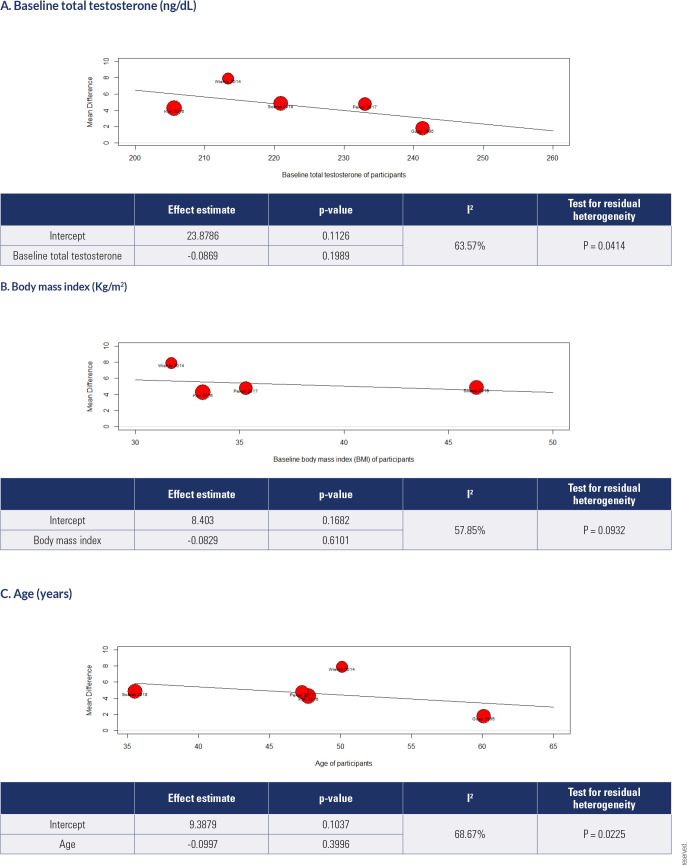
Meta-regression for SERM therapy *vs* placebo assessing the impact of patients’ **A.** Baseline total testosterone; **B.** Body mass index; and **C.** Age on the follicle-stimulating hormone endpoint.

**Figure S10:**
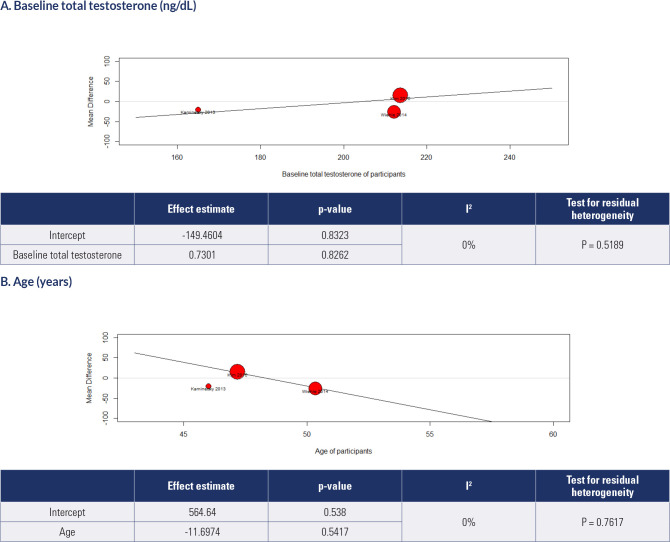
Meta-regression for SERM therapy *vs* testosterone gel assessing the impact of patients’ **A.** Baseline total testosterone and **B.** Age on the total testosterone endpoint.

**Figure S11:**
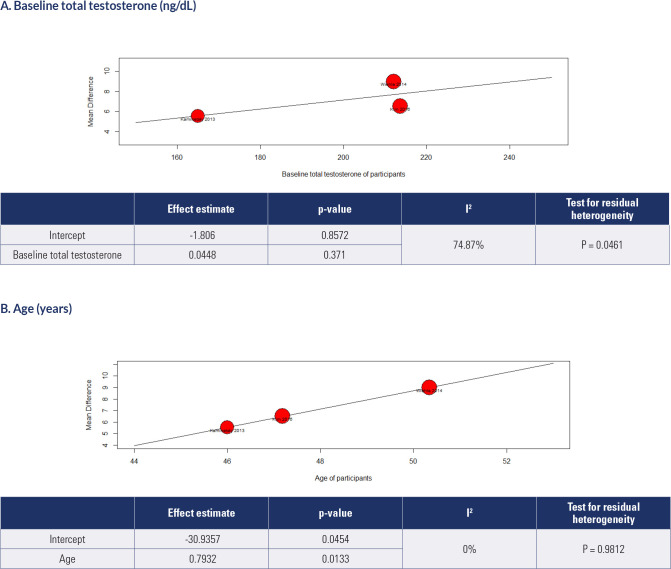
Meta-regression for SERM therapy *vs* testosterone gel assessing the impact of patients’ **A.** Baseline total testosterone and **B.** Age on the luteinizing hormone endpoint.

**Figure S12:**
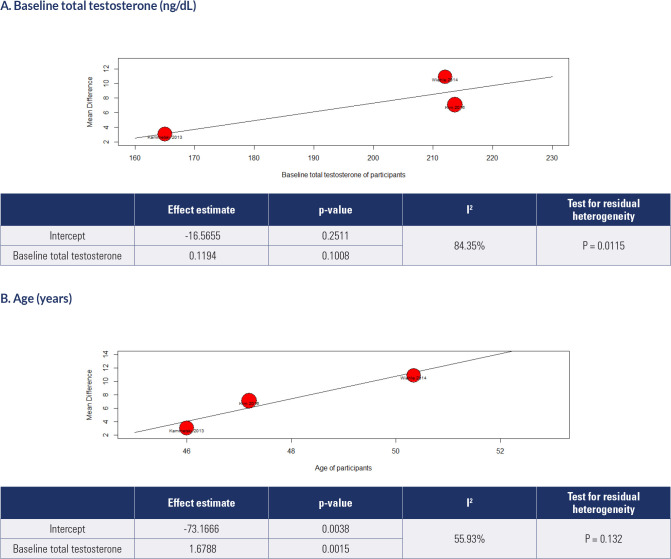
Meta-regression for SERM therapy *vs* testosterone gel assessing the impact of patients’ **A.** Baseline total testosterone and **B.** Age on the follicle-stimulating hormone endpoint.

The benefit of SERM therapy compared to placebo on TT was diminished by advanced age (*p* = 0.0431) and BMI (*p* = 0.0008). There were no significant interactions between baseline TT, age, or BMI on LH and FSH endpoints. The benefit of SERM therapy compared to T gel on LH and FSH was enhanced by advanced age (*p* = 0.0133 and *p* = 0.0015, respectively). However, meta-regression showed no significant interactions between baseline TT or advanced age on the TT endpoint. Additionally, no significant interactions were noted between baseline TT and both LH and FSH endpoints. Meta-regression results are presented in **Supplementary Figures S7-S12**.

### Risk of bias assessment

**Figure 5:**
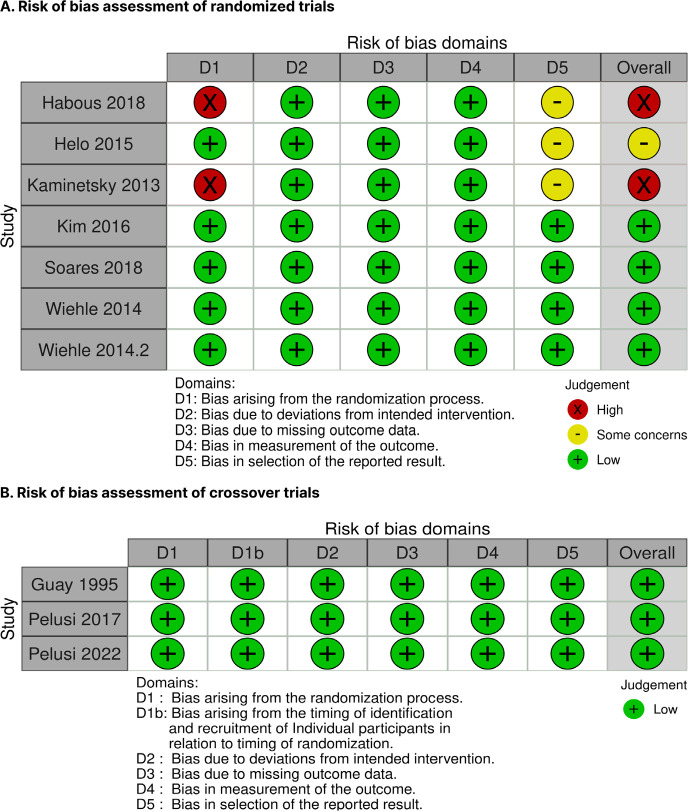
Risk of bias assessment of trials.

The risk of individual within-study bias is depicted in the RoB 2 traffic-light diagram (**Figure 5**). Seven studies were classified as having a low risk of bias ^(11,16-19,21,22)^. Meanwhile, one study was classified with some concerns regarding bias due to issues in the selection of the reported result ^(20)^, and two studies were found to have a high risk of bias due to flaws in the randomization process ^(14,15)^.

### GRADE certainty levels

The endpoints of TT, LH, and FSH were classified as moderate-quality evidence for SERM *vs.* placebo due to wide confidence intervals of the pooled effect estimates and moderate to high heterogeneity. Similarly, TT was classified as moderate-quality evidence for SERM *vs.* T gel, owing to the high risk of bias identified in one study ^(15)^. The endpoints of LH and FSH for SERM *vs.* T gel were categorized as moderate and low-quality evidence, respectively, because of the high risk of bias of one study ^(15)^, coupled with moderate to high heterogeneity and wide confidence intervals of the pooled effect estimates. The TT was categorized as low-quality evidence for SERM *vs.* hCG, due to the high risk of bias inherent in the only RCT that reported this endpoint ^(14)^. A GRADE summary of findings table is provided in **Supplementary Table S4**.

### Trial sequential analyses

**Figure S13:**
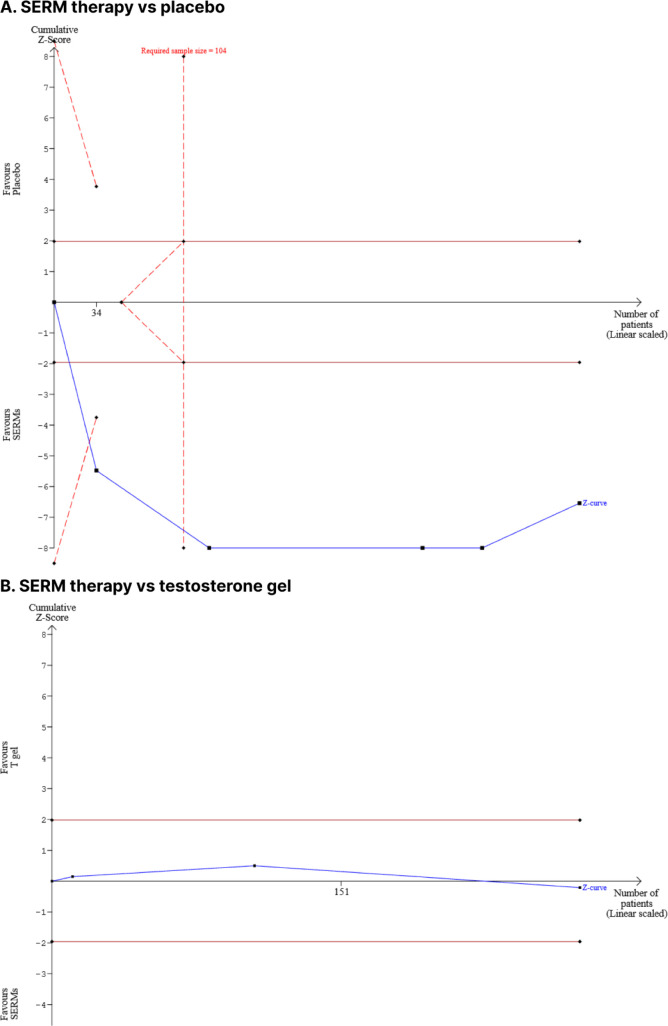
Trial sequential analysis for the total testosterone endpoint. **A.** SERM therapy vs placebo; **B.** SERM therapy *vs* testosterone gel.

**Figure S14:**
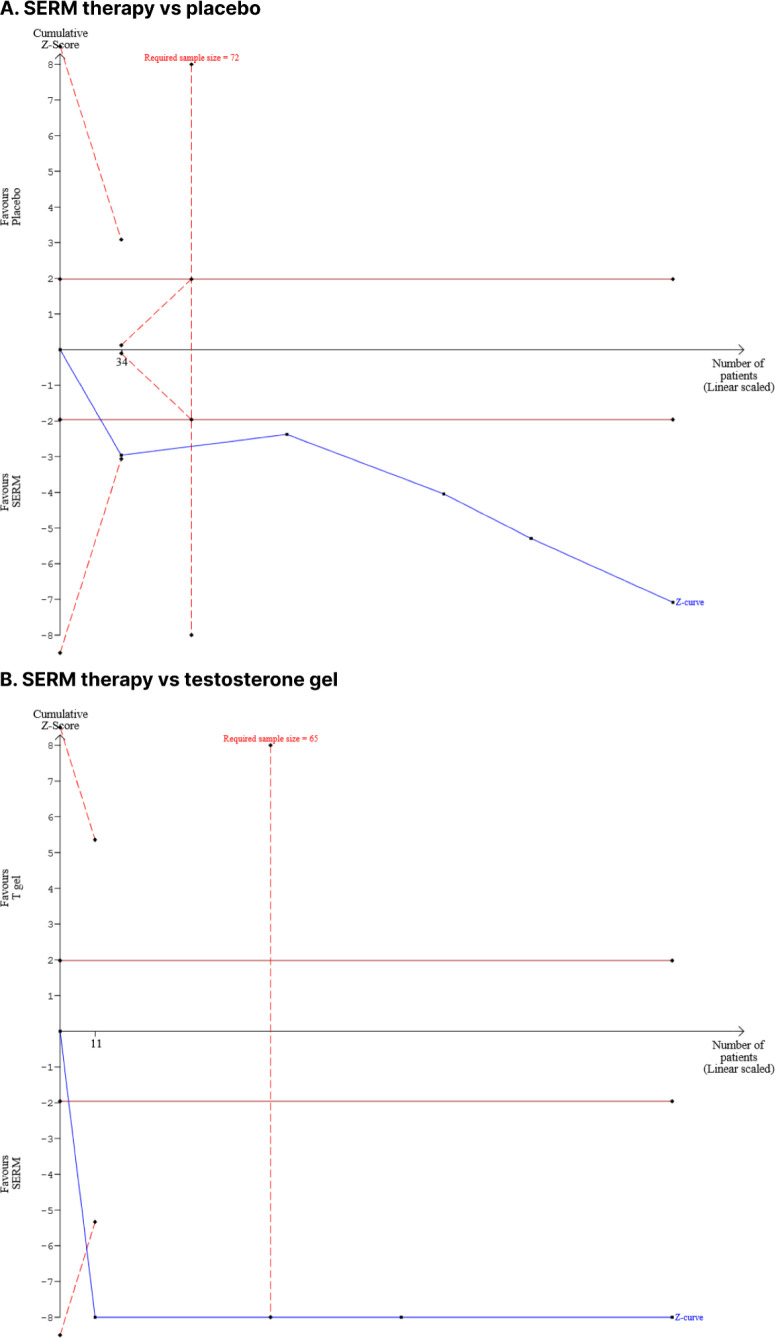
Trial sequential analysis for the luteinizing hormone endpoint. **A.** SERM therapy vs placebo; **B.** SERM therapy *vs* testosterone gel.

**Figure S15:**
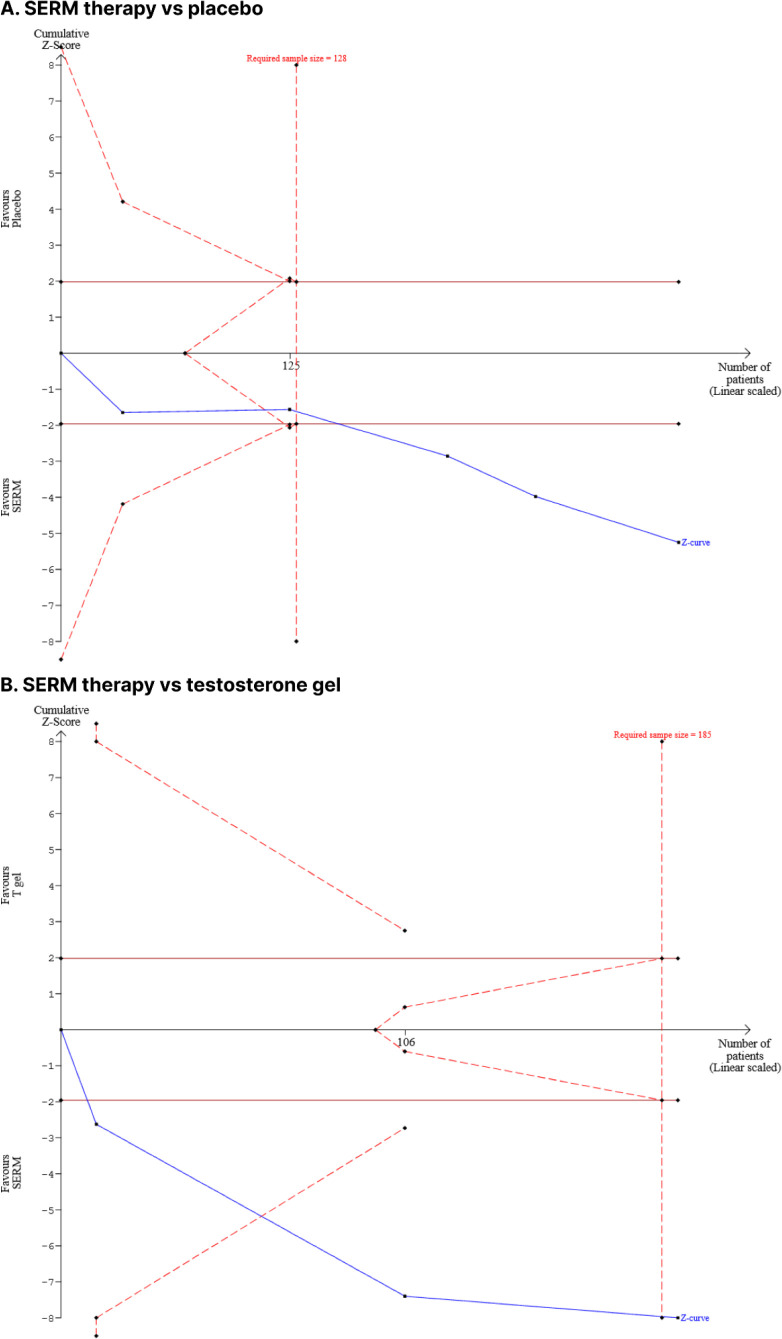
Trial sequential analysis for the follicle-stimulating hormone endpoint. **A.** SERM therapy vs placebo; **B.** SERM therapy *vs* testosterone gel.

Trial sequential analysis showed that enough evidence exists for the benefit of SERM therapy over placebo regarding improvement in TT, LH, and FSH. Also, TSA demonstrated that enough evidence is available for the benefit of SERM therapy over T gel to improve LH and FSH, whereas TT remained comparable between groups. The trial sequential graphs are detailed in **Supplementary Figures S13-S15**.

## DISCUSSION

In this comprehensive systematic review and meta-analysis of 10 RCTs, we compared SERM therapy with placebo, T gel, and hCG for the treatment of men with hypogonadism. Our findings indicated that the use of clomiphene citrate or enclomiphene is associated with a significant increase in TT levels by 273.76 ng/dL compared to placebo, alongside a non-significant difference when compared to the standard topical treatment with T gel. Additionally, SERM treatment significantly improved LH and FSH levels compared to both placebo and T gel, without substantial adverse events.

The effects of SERM therapy were observed in sperm parameters when compared to both placebo and T gel. Current treatment guidelines for male hypogonadism do not recommend the use of testosterone in men who are planning to maintain fertility due to its adverse effects on semen parameters ^(33)^. SERM therapy did not differ from placebo in terms of altering sperm concentration. However, it resulted in a greater increase from baseline compared to T gel. This positive effect on semen parameters has been documented in other systematic reviews and meta-analyses ^(34,35)^. It is based on blocking the negative feedback of E2 in the hypothalamus, leading to increased LH and testosterone production, thereby restoring hormone levels and promoting or preserving spermatogenesis.

While there is ongoing debate about the estrogen receptor-modulating effects of SERMs on plasma estrogen levels and potential adverse outcomes ^(36)^, our analysis found that, compared to placebo and to a lesser extent TRT, SERM therapy led to an increase in plasma estrogen levels. This increase, nonetheless, did not adversely affect sexual function, with no studies reporting a decrease in sexual desire or penile erections. Furthermore, higher estrogen levels have a theoretical potential to positively impact bone mass since many patients with low testosterone levels may also experience reduced bone mineral density due to the effects of sexual steroids ^(37)^.

The application of SERMs is particularly relevant in men with type 2 diabetes mellitus, metabolic syndrome, or obesity, offering a treatment option for hypogonadism that preserves fertility while assessing the associated risks and benefits ^(19,38)^. This approach has garnered support in light of emerging concepts around functional hypogonadism and its reversible nature ^(39)^. The negative effect of exogenous testosterone therapy on testicular function and its direct impact on reduced fertility has led to the treatment of young men with functional hypogonadism using SERMs ^(40)^.

Although SERM therapy effectively treats functional hypogonadism, its neutral effects on glucose, insulin, glycated hemoglobin, and BMI underscore the inadvisability of using testosterone-increasing therapies as a treatment for dysglycemia or obesity ^(8)^.

Furthermore, individuals with obesity and male obesity secondary hypogonadism exhibit low-grade systemic inflammation, a condition exacerbated by the other’s progression ^(41)^. Addressing low testosterone levels can positively influence adherence to healthy lifestyle changes, weight reduction efforts, and overall metabolic health ^(42)^.

Beyond these conditions, the potential therapeutic applications of SERMs are expanding. For instance, in cases of functional hypogonadism associated with Relative Energy Deficiency in Sport, which often leads to diminished sex hormone levels ^(43)^. Another notable indication for SERMs is secondary hypogonadism resulting from anabolic steroid use ^(44)^. In these cases, SERMs offer considerable promise in restoring physiological testosterone. Importantly, their administration is not linked to testosterone spikes or anabolic effects, thereby contributing to health improvements ^(16)^. Furthermore SERMs may also reduce steroid dependence by reversing suppression of the pituitary-gonadal axis and restoring fertility, either alone or in combination with other treatments, such as hCG and aromatase inhibitors ^(14)^.

The temporary use of SERMs may be warranted until the underlying cause of functional hypogonadism is resolved. This is an important difference from treatment with exogenous testosterone replacement therapy performed in patients with organic hypogonadism, which requires ongoing and effective and prolonged treatment to maintain circulating TT levels within a targeted range ^(45)^. A retrospective review examining the use of clomiphene citrate in hypogonadal patients for up to seven years found that over 80% of men maintained TT levels above 450 ng/dL, with 78% reporting subjective improvements in hypogonadism-related symptoms and only 9% experiencing side effects, none of which were significant ^(46)^.

Trial sequential analyses provide strong evidence of benefit for an intervention when the z-curve crosses the trial sequential monitoring boundary and reaches the required sample size ^(32)^. The significant benefits of SERM therapy over placebo for TT, in meta-analyses of TT, LH, and FSH levels were verified at the 90% confidence level through TSA. Similarly, TSA confirmed the advantages of SERM therapy over T gel for LH and FSH levels, though not for TT levels, which remained inconclusive. In choosing a therapy, the possibility of oral therapy, cost, accessibility, and the low probability of changes in hematocrit and PSA levels must be considered. Further RCTs are expected to provide further insight into the efficacy and safety of SERM compared to standard TRTs, whether transdermal or intramuscular.

This study must be interpreted in light of its limitations. First, the cross-over design of two trials introduced a potential unit-of-analysis error into this meta-analysis. This type of error results in a wider CI, reduces the risk of a Type I error, and provides a more conservative estimate of the treatment effect ^(47)^.

Second, certain endpoints exhibited high between-study heterogeneity, such as TT. Nevertheless, we conducted leave-one-out sensitivity analyses and observed consistent results. Moreover, meta-regression demonstrated a significant interaction between the analyzed covariates (age, baseline TT, and BMI) and the effect estimates, which accounts for certain aspects of the observed heterogeneity. Finally, although this study represents the largest pooled analysis of patients treated with clomiphene or enclomiphene, it remains underpowered with regard to safety endpoints.

In conclusion, this systematic review and meta-analysis found that SERM therapy significantly improved TT, LH, and FSH levels in men with hypogonadism compared to placebo, and notably increased LH and FSH levels compared with T gel. These findings suggest that SERM therapy effectively raises TT levels in men with functional hypogonadism and should be considered a viable alternative to T gel therapy.

## Data Availability

datasets related to this article will be available upon request to the corresponding author.

## SUPPLEMENTARY MATERIAL

### I - Search strategy

**Table S1:** Search strategy

PubMed
(Klostilbegit OR Clostilbegit OR Clomid OR Clomide OR Dyneric OR Serophene OR Gravosan OR “Clomiphene Hydrochloride” OR “Hydrochloride, Clomiphene” OR Clomifene OR Chloramiphene OR Clomifen OR “Clomiphene Citrate” OR “Citrate, Clomiphene” OR clomiphene OR enclomiphene) AND (males OR men OR boys OR male) AND (“Hypogonadism, Hypergonadotropic” OR “Hypergonadotropic Hypogonadism” OR “Hypogonadism, Hypogonadotropic” OR “Hypogonadism, Isolated Hypogonadotropic” OR “Hypogonadotropic Hypogonadism” OR hypogonadism)
Embase
(TITLE-ABS-KEY(Klostilbegit) OR TITLE-ABS-KEY(Clostilbegit) OR TITLE-ABS-KEY(Clomid) OR TITLE-ABS-KEY(Clomide) OR TITLE-ABS-KEY(Dyneric) OR TITLE-ABS-KEY(Serophene) OR TITLE-ABS-KEY(Gravosan) OR TITLE-ABS-KEY(“Clomiphene Hydrochloride”) OR TITLE-ABS-KEY(“Hydrochloride, Clomiphene”) OR TITLE-ABS-KEY(Clomifene) OR TITLE-ABS-KEY(Chloramiphene) OR TITLE-ABS-KEY(Clomifen) OR TITLE-ABS-KEY(“Clomiphene Citrate”) OR TITLE-ABS-KEY(“Citrate, Clomiphene”) OR TITLE-ABS-KEY(clomiphene) OR TITLE-ABS-KEY(enclomiphene)) AND (TITLE-ABS-KEY(males) OR TITLE-ABS-KEY(men) OR TITLE-ABS-KEY(boys) OR TITLE-ABS-KEY(male)) AND (TITLE-ABS-KEY(“Hypogonadism, Hypergonadotropic”) OR TITLE-ABS-KEY(“Hypergonadotropic Hypogonadism”) OR TITLE-ABS-KEY(“Hypogonadism, Hypogonadotropic”) OR TITLE-ABS-KEY(“Hypogonadism, Isolated Hypogonadotropic”) OR TITLE-ABS-KEY(“Hypogonadotropic Hypogonadism”) OR TITLE-ABS-KEY(hypogonadism))
Cochrane Library
((Klostilbegit):ti,ab,kw OR (Clostilbegit):ti,ab,kw OR (Clomid):ti,ab,kw OR (Clomide):ti,ab,kw OR (Dyneric):ti,ab,kw OR (Serophene):ti,ab,kw OR (Gravosan):ti,ab,kw OR (“Clomiphene Hydrochloride”):ti,ab,kw OR (“Hydrochloride, Clomiphene”):ti,ab,kw OR (Clomifene):ti,ab,kw OR (Chloramiphene):ti,ab,kw OR (Clomifen):ti,ab,kw OR (“Clomiphene Citrate”):ti,ab,kw OR (“Citrate, Clomiphene”):ti,ab,kw OR (clomiphene):ti,ab,kw OR (enclomiphene):ti,ab,kw) AND ((males):ti,ab,kw OR (men):ti,ab,kw OR (boys):ti,ab,kw OR (male):ti,ab,kw) AND ((“Hypogonadism, Hypergonadotropic”):ti,ab,kw OR (“Hypergonadotropic Hypogonadism”):ti,ab,kw OR (“Hypogonadism, Hypogonadotropic”):ti,ab,kw OR (“Hypogonadism, Isolated Hypogonadotropic”):ti,ab,kw OR (“Hypogonadotropic Hypogonadism”):ti,ab,kw OR (hypogonadism):ti,ab,kw)
Web of Science
(TS=(Klostilbegit) OR TS=(Clostilbegit) OR TS=(Clomid) OR TS=(Clomide) OR TS=(Dyneric) OR TS=(Serophene) OR TS=(Gravosan) OR TS=(“Clomiphene Hydrochloride”) OR TS=(“Hydrochloride, Clomiphene”) OR TS=(Clomifene) OR TS=(Chloramiphene) OR TS=(Clomifen) OR TS=(“Clomiphene Citrate”) OR TS=(“Citrate, Clomiphene”) OR TS=(clomiphene) OR TS=(enclomiphene)) AND (TS=(males) OR TS=(men) OR TS=(boys) OR TS=(male)) AND (TS=(“Hypogonadism, Hypergonadotropic”) OR TS=(“Hypergonadotropic Hypogonadism”) OR TS=(“Hypogonadism, Hypogonadotropic”) OR TS=(“Hypogonadism, Isolated Hypogonadotropic”) OR TS=(“Hypogonadotropic Hypogonadism”) OR TS=(hypogonadism))

### II - Study features

**Table S2:** Inclusion and exclusion criteria of included studies

Study	Inclusion criteria	Exclusion criteria
Guay 1995	Men with secondary hypogonadism. Erectile dysfunction for 6 or more months. Low serum free testosterone levels and normal (or unstimulated) serum gonadotropin levels in an early (0800-1000 h) sample. Serum total testosterone ≤275 ng/dL. Normal results on magnetic resonance image studies of the hypothalamic-pituitary axis. Normal levels of serum prolactin, estradiol, and sex hormone-binding globulin.	
Habous 2018	Men with low serum testosterone levels on at least two samples (≤300 ng/dL). Three or more positive symptoms in the qADAM questionnaire.	Primary testicular failure Hypogonadotropic hypogonadism. Prior history of testosterone therapy. Chromosomal abnormalities, Cryptorchidism history, or a single testis.
Helo 2015	Male infertility (defined as the inability to conceive after 1 year). Hypogonadism (defined as serum T less than 350 ng/dL and LH between 1.2 and 8.6 mIU/ mL). Males aged 18 - 50 years. Baseline morning TT between 150 and 350 ng/dL on two consecutive morning measurements 1 week apart.	Sperm count <1 million. BMI >40 kg/m^2^. Hematocrit <36% or >52%. History of prostate-specific antigen >4.0 ng/dL. History of chronic opioid use, intravenous or inhaled steroid use within the previous 3 months, or use of drugs known to affect steroid hormone or sex hormone-binding globulin levels. Testicular or pituitary disease. History of prostate cancer or severe benign prostatic hypertrophy.
Kaminetsky 2013	Men taking topical testosterone therapy for at least 6 months before the screening visit. Secondary hypogonadism. Total testosterone <300 ng/dL.	History of idiopathic infertility due to primary hypogonadism, testicular failure, Kallmann syndrome, or any other infertility condition. Any clinically significant medical condition rendering the subjects infertile or marginally fertile Usage of testosterone, androgen, estrogen, or other hormone products before enrollment in the study. Men unwilling to discontinue taking the product for at least 30 days before receiving study medication. Substance abuse at screening.
Kim 2016	Overweight (BMI 25 - 42 kg/m^2^ inclusive). Males aged 18 to 60 years. Secondary hypogonadism. At least 2 consecutive morning testosterone assessments <300ng/dL, one of which confirmed at baseline. LH <9.4 mIU/mL. Sperm concentration ≥15 million/mL at visit 2 and baseline. Ability to complete the study in compliance with the protocol. Ability to understand and provide written informed consent. Agreement to provide a total of at least 4 semen samples in a sponsor-approved clinic on 4 separate occasions.	Prior use of testosterone therapy within the last 6 months. Use of spironolactone, cimetidine, Clomid, 5α-reductase inhibitors, hCG, androgen, estrogen, anabolic steroid, DHEA, or herbal hormone products during the study. Use of Clomid in the past year. Any clinically significant laboratory abnormality with no prior written sponsor approval. Uncontrolled hypertension or diabetes mellitus based on the investigator’s assessment at baseline. Hematocrit >54. Use of an investigational drug or product, or participation in a drug or medical device research study within 30 days before receiving the study medication. Known hypersensitivity to Clomid. Nuclear sclerosis cataract or cortical cataract grade >2 based on a 0-4 scale or evidence of posterior subcapsular cataract. Abnormal fundoscopy exam (e.g., central retinal vein occlusion). Any condition that would interfere with the participant’s ability to provide informed consent, comply with study instructions, possibly confound interpretation of study results, or endanger the participant if they took part in the study. Diagnosis of irreversible infertility or compromised fertility (cryptorchism, Kallman Syndrome, primary hypogonadism, vasectomy, or tumors of the pituitary), or history of evaluation or treatment for low fertility. Current or history of breast cancer. Current or history of prostate cancer or a suspicion of prostate disease unless ruled out by prostate biopsy, or PSA>3.6. Presence or history of hyperprolactinemia (prolactin >20 ng/mL). Chronic use of medications such as glucocorticoids (chronic use of inhaled or topical glucocorticoids are acceptable). History of drug abuse or chronic narcotic use including methadone. Recent history of alcoholism or illegal substance or steroid abuse (<2 years) or moderate alcohol use (>21 drinks per week). Subjects with a history of HIV and/or Hepatitis C. Subjects with end-stage renal disease. History of liver disease (including malignancy) or confirmed aspartate aminotransferase or alanine aminotransferase >3 times the upper limit of normal. History of clinically relevant myocardial infarction (within the previous year), unstable angina, symptomatic heart failure, ventricular dysrhythmia, or history of QTc interval prolongation. History of clinically relevant cerebrovascular disease. History of venous thromboembolic disease (e.g. deep vein thrombosis or pulmonary embolism). History of erythrocytosis or polycythemia. Subjects unable to provide a semen sample in a sponsor-approved clinic. Enrollment in a previous Androxal study. Subjects with Type I Diabetes.
Pelusi 2017^a^	Males aged 35 - 55 years. BMI >30 kg/m^2^. Serum total testosterone levels ≤3 ng/mL (measured by electrochemiluminescence immunoassay) New diagnosis of impaired glucose tolerance or Type II Diabetes under criteria of the American Diabetes Association with glycated hemoglobin <8.5%.	Hypogonadism due to organic or genetic causes at hypothalamic-pituitary or gonadal levels detected with clinical, hormonal, and radiological examinations, with no need for genetic tests owing to their clinical history. Impaired glucose tolerance or Type II Diabetes patients, already on anti-diabetic treatment. Medication interfering with glucose metabolism, including steroid treatment. Any acute or chronic illness that would contraindicate the use of the study medications.
Pelusi 2022^a^	Males aged 35 - 55 years. BMI >30 kg/m^2^. Serum total testosterone levels ≤3 ng/mL (measured by electrochemiluminescence immunoassay) New diagnosis of impaired glucose tolerance or Type II Diabetes under criteria of the American Diabetes Association with glycated hemoglobin <8.5%. Agreement to fulfill the IIEF-15 and the qADAM questionnaires at all four-time points.	Hypogonadism due to organic or genetic causes at hypothalamic-pituitary or gonadal levels detected with clinical, hormonal, and radiological examinations, with no need for genetic tests owing to their clinical history. Impaired glucose tolerance or Type II Diabetes patients, already on anti-diabetic treatment. Medication interfering with glucose metabolism, including steroid treatment. Any acute or chronic illness that would contraindicate the use of the study medications. Being unable or refusing to fulfill the IIEF-15 and the qADAM questionnaires at all four-time points.
Soares 2018	Community-dwelling men. Secondary hypogonadism. 20-50 years. BMI >30 kg/m^2^. Morning TT ≤300 ng/dL repeated twice at least 1 week apart. Symptoms assessed with the qADAM questionnaire. Low or inappropriately normal LH ^(^(reference range 1.7-8.6 IU/L).	Serious systemic diseases (e.g. heart failure NYHA III and IV, liver disease, or renal insufficiency). Strenuous physical exercise practice. Use of certain medications (e.g. opioids or methadone) or chronic steroid use. Eating disorders. Addictive drugs. Testicular volume <4 mL. Hyperprolactinemia. Hemochromatosis. Intermittent or previous smoking history. No abnormalities of the pituitary gland on nuclear magnetic resonance.
Wiehle 2014	Healthy males aged 21 - 65 years. All clinical laboratory tests within normal ranges. Secondary hypogonadism Morning total testosterone <250ng/dL (two assessments at least 10 days apart). Ability to complete the study in compliance with the protocol. Ability to understand and provide written informed consent. Agreement to use double barrier contraception if with a fertile female partner. Agreement to provide a semen sample in the clinic.	Use of injectable, oral, topical, or subcutaneous pelleted testosterone within 6 months before study. Use of spironolactone, cimetidine, Clomid, 5α-reductase inhibitors, hCG, androgen, estrogen, anabolic steroid, DHEA, or herbal hormone products during the study. Use of Clomid in the past year. Uncontrolled hypertension or diabetes mellitus based on the Investigator’s assessment at baseline. Subjects treated for Type II diabetes with glycemic control were allowed into the study. Hematocrit >50%. Hemoglobin >17 g/dL. Clinically significant abnormal findings on screening examination. Use of an investigational drug or product, or participation in a drug or medical device research study within 30 days before receiving study medication. Known hypersensitivity to Clomid. Nuclear sclerosis cataract or cortical cataract grade >2 based on a 0-4 scale or any trace of posterior subcapsular cataract. Any condition which in the opinion of the investigator would interfere with the participant’s ability to provide informed consent, comply with study instructions, possibly confound interpretation of study results, or endanger the participant if he took part in the study.
Wiehle 2014		Irreversibly infertile or compromised fertility (cryptorchism, Kallman Syndrome, primary hypogonadism, vasectomy, or tumors of the pituitary). Current or history of breast cancer. Current or history of prostate cancer or a suspicion of prostate disease unless ruled out by prostate biopsy, or a PSA >3.6. Presence or history of hyperprolactinemia. Chronic use of medications (e.g., glucocorticoids). Cystic fibrosis (mutation of the CFTR gene). Subjects unable to provide a semen sample in the clinic. BMI >36 kg/m^2^.
Wiehle 2014.2	Males aged 18 -75 years. Secondary hypogonadism (TT <350ng/dL). FSH within 1.5 to 12.4 IU/L. LH within 1.7 to 8.6 IU/L. Ability to complete the study in compliance with the protocol. Provide written informed consent.	

**Footnotes: a:** overlapping population. **Abbreviations:** BMI, body mass index; DHEA, Dehydroepiandrosterone; FSH, follicle-stimulating hormone; hCG, human chorionic gonadotropin; IIEF, International Index of Erectile Function; LH, luteinizing hormone; NYHA, New York Heart Association; PSA, prostate-specific antigen; qADAM, quantitative Androgen Deficiency in Aging Males; TT, total testosterone.

**Table S3:** Additional characteristics of included studies

Study	Intervention/ Control	PSA, mg/dL, SG/CG	Estradiol, pg/mL, SG/CG	SHBG, nmol/L, SG/CG	FT, pg/mL, SG/CG	HbA1c (%), SG/CG	ADAM questionnaire, SG/CG	Time since hypogonadism diagnosis, years, SG/CG
Guay 1995	Clomiphene citrate 50 mg/ Placebo	NA	18±8.1/ 18±8.1	1±0.3/ 1±0.3^a^	9.9±2.4/ 9.9±2.4	NA	NA	4.0±3.6/ 4.0±3.6
Habous 2018	Clomiphene citrate 50 mg/ 5000IU hCG injections	NA	NA	NA	NA	6.5±1.9	20.5±3.8	NA
Helo 2015	Clomiphene citrate 25mg/ Anastrozole 1 mg/	NA	27.6±0.9/ 26.7±0.9	22±8.3/ 23±8.5	8.3±0.9/ 9.3±0.9	NA	37±1.9/ 36±1.6	1.3±0.5/ 1.9±1.4
Kaminetsky 2013	Enclomiphene citrate 25 mg/ T gel	NA	10.5±2.5/ 10±0	20.3±8.0/ 16.6±4.9	NA	NA	NA	1.8
Kim 2016	Enclomiphene citrate 12.5 mg/ Enclomiphene citrate 25 mg/ T gel 1.62%/ Placebo	NA	NA	NA	NA	NA	NA	NA
Pelusi 2017^b^	Clomiphene citrate 25 mg + metformin 2 g/ Placebo + metformin 2 g	NA	23.3±7.8/ 23.3±7.8	22.8±11.1/ 22.8±11.1	74.4±22.9/ 74.4±22.9	5.96±0.62/ 5.96±0.62	NA	NA
Pelusi 2022^b^	Clomiphene citrate 25 mg + metformin 2 g/ Placebo + metformin 2 g	NA	24.4±7.3/ 24.4±7.3	21.5±8.0/ 21.5±8.0	68.1±15/ 68.1±15	6.0±0.7/ 6.0±0.7	32.9±5.3/ 32.9±5.3	NA
Soares 2018	Clomiphene citrate 50 mg/ Placebo	0.62±0.41/ 0.58±0.50	32.5/ 12.6/ 33.5±12.6	21.6±7.9/ 22.3±9.1	55.2±17.3/ 55.8±19.4	5.79±0.83/ 5.73±0.70	5.2±2.6/ 5.0±2.1^c^	NA
Wiehle 2014	Enclomiphene citrate 12.5 mg/ Enclomiphene citrate 25 mg/ T gel 1%/ Placebo	NA	20.8±12.4/ 24.7±15.9/ 26.3±21.8/ 22.3±15	24.7±12.9/ 26.4±12.1/ 26.1±10.5/ 27.7±15.5	NA	NA	NA	NA
Wiehle 2014.2	Enclomiphene citrate 12.5 mg/ Enclomiphene citrate 25 mg/ Enclomiphene citrate 50 mg/ T gel 5 g/ T gel 10 g/ Placebo	NA	23.2±6.3/ 22.6±4.5/ 25.4±8.4/ 24.6±11.4/ 27.6±17.1/ 17.4±4.1	19.7±6.0/ 24.9±7.8/ 22.3±11.2/ 19.6±10.7/ 17.8±8.5/ 22.4±10.3	NA	NA	NA	NA

**Footnotes:** data are means ± SD or (range). a: ucg DHT bound/dL; b: overlapping population; c: positive responses in the ADAM questionnaire. **Abbreviations:** ADAM, Androgen Deficiency in Aging Men; CG, control group; FT, free testosterone; HbA1c, glycated hemoglobin; NA, not available; PSA, prostate-specific antigen; SD, standard deviation; SG, Selective estrogen receptor modulator (SERM) group; SHBG, sex hormone-binding globulin.

### III - GRADE assessment

**Table S4** GRADE summary of findings table for primary endpoints. **A.** Selective estrogen-receptor modulator (SERM) therapy vs placebo; **B.** SERM therapy vs testosterone gel; **C.** SERM therapy vs hCG

#### A. SERM therapy vs placebo

**Table:**

Certainty assessment							N^o^ of patients		Effect	Certainty	Importance
N^o^ of studies	Study design	Risk of bias	Inconsistency	Indirectness	Imprecision	Other considerations	SERM therapy	placebo	Absolute (95% CI)		
Total testosterone (follow-up: range 2 months to 4 months; assessed with: Mean difference)											
5	randomised trials	not serious	very serious^a^	not serious	serious^b^	very strong association	227	195	MD **273.76 ng/dL higher** (191.87 higher to 355.66 higher)	⨁⨁⨁◯ Moderate	CRITICAL
**Luteinizing hormone (follow-up: range 2 months to 4 months; assessed with: MD)**											
5	randomised trials	not serious	serious^c^	not serious	serious^b^	strong association	183	154	MD **4.66 UI/L higher** (3.37 higher to 5.94 higher)	⨁⨁⨁◯ Moderate	CRITICAL
**Follicle stimulating hormone (follow-up: range 2 months to 4 months; assessed with: Mean difference)**											
5	randomised trials	not serious	serious^c^	not serious	serious^b^	strong association	183	154	MD **4.59 UI/L higher** (2.88 higher to 6.3 higher)	⨁⨁⨁◯ Moderate	CRITICAL

#### B. SERM therapy vs testosterone gel

**Table:**

Certainty assessment							N^o^ of patients		Effect	Certainty	Importance
N^o^ of studies	Study design	Risk of bias	Inconsistency	Indirectness	Imprecision	Other considerations	SERM therapy	testosterone gel	Absolute (95% CI)		
Total testosterone (follow-up: range 3 months to 6 months; assessed with: mean difference)											
3	randomised trials	serious^d^	not serious	not serious	not serious	none	183	154	MD **5.41 ng/dL higher** (43.44 lower to 54.27 higher)	⨁⨁⨁◯ Moderate	CRITICAL
**Luteinizing hormone (follow-up: range 3 months to 6 months; assessed with: mean differenc**e)											
3	randomised trials	serious^d^	serious^c^	not serious	serious^b^	very strong association	109	81	MD **7.13 UI/L higher** (5.12 higher to 9.13 higher)	⨁⨁⨁◯ Moderate	CRITICAL
**Follicle stimulating hormone (follow-up: range 3 months to 6 months; assessed with: mean differenc**e)											
3	randomised trials	serious^d^	very serious^a^	not serious	serious^b^	very strong association	109	81	MD **6.98 UI/L higher** (3.04 higher to 10.93 higher)	⨁⨁◯◯ Low	CRITICAL

#### C. SERM therapy vs hCG

**Table:**

Certainty assessment							N^o^ of patients		Effect	Certainty	Importance
N^o^ of studies	Study design	Risk of bias	Inconsistency	Indirectness	Imprecision	Other considerations	SERM therapy	hCG	Absolute (95% CI)		
**Total testosterone (follow-up: mean 3 months)**											
1	randomised trials	very serious^e-^	not serious	not serious	not serious	none	95	94	MD 23.36 ng/dL higher (9.33 higher to 37.39 higher)	⨁⨁◯◯ Low	CRITICAL

**CI:** confidence interval; MD: mean difference

#### Explanations

**a.** High heterogeneity

**b.** Wide confidence interval

**c**. Moderate heterogeneity

**d.** One study with a high risk of bias

**e**. Study with a high risk of bias
